# Identification of key biomarkers of the glomerulus in focal segmental glomerulosclerosis and their relationship with immune cell infiltration based on WGCNA and the LASSO algorithm

**DOI:** 10.1080/0886022X.2023.2202264

**Published:** 2023-04-25

**Authors:** Yun Xia Zhang, Juan Lv, Jun Yuan Bai, XiaoWei Pu, En Lai Dai

**Affiliations:** a College of Integrated Traditional and Western Medicine, Gansu University of Traditional Chinese Medicine, Lanzhou, China; b Gansu Provincial Hospital of Traditional Chinese Medicine, Lanzhou, China; c Affiliated Hospital of Gansu University of Chinese Medicine, Lanzhou, China

**Keywords:** Focal segmental glomerulosclerosis, weighted gene coexpression network analysis, least absolute shrinkage and selection operator, immune cell infiltration, TGFB1, NOTCH1

## Abstract

**Objective:**

The aim of our study was to identify key biomarkers of glomeruli in focal glomerulosclerosis (FSGS) and analyze their relationship with the infiltration of immune cells.

**Methods:**

The expression profiles (GSE108109 and GSE200828) were obtained from the GEO database. The differentially expressed genes (DEGs) were filtered and analyzed by gene set enrichment analysis (GSEA). MCODE module was constructed. Weighted gene coexpression network analysis (WGCNA) was performed to obtain the core gene modules. Least absolute shrinkage and selection operator (LASSO) regression was applied to identify key genes. ROC curves were employed to explore their diagnostic accuracy. Transcription factor prediction of the key biomarkers was performed using the Cytoscape plugin IRegulon. The analysis of the infiltration of 28 immune cells and their correlation with the key biomarkers were performed.

**Results:**

A total of 1474 DEGs were identified. Their functions were mostly related to immune-related diseases and signaling pathways. MCODE identified five modules. The turquoise module of WGCNA had significant relevance to the glomerulus in FSGS. TGFB1 and NOTCH1 were identified as potential key glomerular biomarkers in FSGS. Eighteen transcription factors were obtained from the two hub genes. Immune infiltration showed significant correlations with T cells. The results of immune cell infiltration and their relationship with key biomarkers implied that NOTCH1 and TGFB1 were enhanced in immune-related pathways.

**Conclusion:**

TGFB1 and NOTCH1 may be strongly correlated with the pathogenesis of the glomerulus in FSGS and are new candidate key biomarkers. T-cell infiltration plays an essential role in the FSGS lesion process.

## 1. Introduction

Nephrotic syndrome (NS) is a manifestation of glomerular disease that is characterized by severe albuminuria with hypoproteinemia and edema or hyperlipidemia. The first-line drug for the clinical treatment of NS is steroid hormones, so NS can be divided into steroid-sensitive, steroid-dependent, and steroid-resistant types according to the response to intensive steroid therapy. Most steroid-resistant NS (SRNS) patients do not have a specific therapeutic strategy and are prone to end-stage renal disease. In these patients, most pathological manifestations are focal segmental glomerulosclerosis (FSGS). The pathological characteristics of FSGS are mainly podocyte hypoplasia, podocyte death, glomerular basement membrane exposure, capillary collapse, etc. [1]. FSGS is divided into primary and secondary FSGS. In primary FSGS, glomerular sclerosis is related to podocyte abnormalities, and most of the podocytes are directly damaged. Recently, studies have shown that primary FSGS is associated with causative circulating permeable factors (soluble urokinase plasminogen activator receptor (suPAR)) [2,3]. However, in secondary FSGS, the causes of podocyte damage are mostly indirect, such as obesity, reduced kidney mass, drug toxicity, viral infection, and other secondary factors [4]. FSGS can occur in a variety of populations. The incidence of FSGS in children and adults with NS is 7–10% and 20–30%, respectively [5]. Due to immune alterations in a proinflammatory or profibrotic environment, some patients with pathological features of FSGS do not respond clinically to immunotherapy, ultimately leading to end-stage renal disease [6]. It is crucial to research the connection between the infiltration of immune cells and FSGS lesions. There are great limitations in the diagnostic methods of FSGS. A deeper exploration of the core markers linked to FSGS and the connection to the infiltration of immune cells may contribute to the development of effective diagnostic and therapeutic methods.

Coexpression analysis is a powerful technique used to construct scale-free gene coexpression networks. Weighted gene coexpression network analysis (WGCNA) is a systems biology technique used to describe the correlation patterns of genes in microarray specimens [7]. WGCNA does not find abnormally expressed gene features. It can assign highly correlated genes to the same coexpression module to identify modules related to a clinical phenotype and more effectively identify diagnostic and therapeutic markers [8]. Least absolute shrinkage and selection operator (LASSO) has been used to construct Cox proportional hazards regression models for diagnosis and prognosis based on transcriptome profiles [9].

Zhu et al. [10] used comprehensive bioinformatics methods to reveal the pathogenesis of FSGS, but WGCNA and immune cells were not involved. Therefore, this paper further identified the potential key biomarkers of FSGS and revealed its pathogenesis from these two aspects.

We used microarray chip data from glomerular samples obtained from FSGS patients and healthy control individuals. Subsequently, WGCNA and LASSO were used to determine the key biomarkers involved in FSGS glomeruli. To understand the functions of key biomarkers more comprehensively and speculate on their potential mechanisms of action, we first predicted their transcription factors. The correlation between key biomarkers and immune cell infiltration was analyzed.

In our study, we validated key biomarkers associated with FSGS glomeruli using WGCNA and LASSO using microarray data of mRNA from FSGS patients and healthy donor glomeruli samples. We predict transcription factors for key biomarkers. Subsequently, the association between key biomarkers and immune cell infiltration was analyzed. The findings may provide unique insights for expanding diagnostic and therapeutic approaches for FSGS.

## 2. Materials & methods

### 2.1. Data collection

We collected the data of GSE200828 and GSE108109 with the help of the GEO (https://www.ncbi.nlm.nih.gov/geo/) database. We chose seventy-three samples, 61 glomerulus samples for the FSGS group and 12 samples for the healthy control group (Table 1). The sequencing platform was GPL19983. The external validation dataset, GSE129973, included 40 samples, 20 FSGS glomerular samples and 20 control samples. GPL17586 was used as the sequencing platform.

**Table 1.** Summary of the included microarray sets.

Eries	Platform	Healthy controls	Glomerulus of FSGS	Species
GSE108109	GPL19983	6 GSM2889865, GSM2889866 GSM2889867, GSM2889868 GSM2889869, GSM2889870	30 GSM2889849,GSM2889850,GSM2889851,GSM2889852 GSM2889853,GSM2889854,GSM2889855,GSM2889856 GSM2889857,GSM2889858,GSM2889859,GSM2889860 GSM2889861,GSM2889862,GSM2889863,GSM2889864 GSM2889898,GSM2889899,GSM2889900,GSM2889901 GSM2889902,GSM2889903,GSM2889904,GSM2889905 GSM2889906,GSM2889907,GSM2889908,GSM2889909 GSM2889910,GSM2889911	Homo sapiens
GSE200828	GPL19983	6 GSM6044635, GSM6044636 GSM6044637, GSM6044638 GSM6044639, GSM6044640	31 GSM6044566,GSM6044567,GSM6044569.GSM6044573 GSM6044574,GSM6044576,GSM6044578,GSM6044587 GSM6044607,GSM6044611,GSM6044614,GSM6044616 GSM6044617,GSM6044625,GSM6044631,GSM6044644 GSM6044645,GSM6044657,GSM6044658,GSM6044660 GSM6044666,GSM6044669,GSM6044670,GSM6044672 GSM6044677,GSM6044680,GSM6044682,GSM6044683 GSM6044686,GSM6044700 GSM6044563	Homo sapiens
GSE129973	GPL17586	20 GSM3728791,GSM3728792 GSM3728793,GSM3728794 GSM3728795,GSM3728796 GSM3728797,GSM3728798 GSM3728799,GSM3728800 GSM3728801,GSM3728802 GSM3728803,GSM3728804 GSM3728805,GSM3728806 GSM3728807,GSM3728808 GSM3728809,GSM3728810	20 GSM3728811,GSM3728812 GSM3728813,GSM3728814 GSM3728815,GSM3728816 GSM3728817,GSM3728818 GSM3728819,GSM3728820 GSM3728821,GSM3728822 GSM3728823,GSM3728824 GSM3728825,GSM3728826 GSM3728827,GSM3728828 GSM3728829,GSM3728830	Homo sapiens

### 2.2. Selection of differentially expressed genes (DEGs)

Our data of GSE108109 and GSE200828 were edited with the ‘sva’ package. The ‘limma’ package of R was applied to purifier our data and identify the DEGs. The ‘pheatmap’ package of R was used to produce heatmaps for the DEGs. The volcano map of DEGs was generated using the ‘ggplot2’ package of R software. Data standardization was performed, and a *p* value <0.05 and log fold change > 1 were used as thresholds [11].

### 2.3. Gene set enrichment analysis (GSEA) of DEGs

The ‘limma’, ‘org.Hs.eg.db’, ‘cluster Profiler’, and ‘enrichplot’ packages were applied for GSEA. ImmuneSigDB provided a useful tool for detecting subtle patterns of similarity and difference in large-scale datasets of gene expression from cells and tissues in the immune system. The ‘immunesigdb’ files were obtained and used for the enrichment analysis of GSEA [12].

### 2.4. Protein–protein interaction (PPI) network creation and hub module analysis

The STRING [13] database was used to build a PPI network for the DEGs. To study the connections among the DEGs, significance was set at a confidence level of >0.4. Cytoscape 3.8.0 [14] was applied to understand the PPI network and subsequent analysis. We used the Molecular Complex Detection (MCODE) plug-in [15] to perform cluster analysis of the DEGs. The conditions for significant modules were as follows: MCODE score >5, number of nodes ≥10, maximum depth = 100, degree cutoff value = 2, node score cutoff value = 0.2, and k-score = 2. Subsequently, we used the Cytoscape plug-in cyto-Hubba [16] to rank the core genes of each module. We used a ‘Maximal Clique Centrality (MCC) algorithm’ [17] to arrange the core genes of each MCODE module in order. The functional enrichment of genes in each MCODE was determined by Gene Ontology (GO) and Kyoto Encyclopedia of Genes and Genomes (KEGG). The analyses were performed using the ‘org.Hs.eg.db’, ‘cluster Profiler’, ‘enrichplot’, and ‘ggplot2’ packages in R [18].

### 2.5. Weighted coexpression network creation

R ‘WGCNA’ was applied to create the coexpression network for the DEGs [19]. The soft threshold parameter (*β*) was provided to assess the strong links among the genes, and an adjacency matrix was created. We use network connectivity to evaluate whether the adjacency matrix can be successfully converted to a topological overlap metric (TOM). We constructed a clustering tree graph for our TOM matrix by means of average linkage hierarchical clustering. The main manifestations were different branches and colors, building module relationships, calculating the link with gene modules and syndromes, and identifying modules linked with the clinical characteristics. We determined the number of genes in the total module. The number of spliced genes per module was no less than 70. The screening criteria for core genes in every module were module membership >0.8 and gene significance >0.5 [20].

### 2.6. Identification of the hub genes

We first obtained the intersecting genes using the ‘Venn’ package of R. Second, intersecting genes were derived from WGCNA core modules and important modules that met the MCODE module screening criteria. We used the ‘glmnet’ of the R package and a LASSO algorithm to decide the end LASSO genes by the intersecting genes [21]. Boxplots were mainly provided to represent the differences in lasso gene expression between the two groups. Using the pROC package in R, we applied ROC (the receiver operating characteristic) and area under the curve (AUC) to assess the diagnostic significance of the core genes. Hub genes were those with an AUC >0.75 [22]. We used an external dataset (GSE129973) to test the diagnostic value for hub genes by boxplot, ROC curve and AUC.

### 2.7. Identification and differential analysis of transcription factors (TFs) acting on hub genes

iRegulon [23] is a plug-in of Cytoscape that is a computational method for identifying master regulators and direct target genes in human genetic signatures, that is, a set of coexpressed genes. The plug-in used the normalized expansion score (NES) to estimate the accuracy of the prediction results. The NES value and reliability were positively correlated. When the NES >4, the transcription factors were applied to construct regulatory networks. Cytoscape was applied to visualize relationships between core genes and TFs. We performed differential analysis of transcription factors with the aid of the R packages ‘limma’ and ‘ggpubr’. The ‘limma’ R software package was used for the difference analysis, and then the graph was plotted using ‘ggpubr’. GO function analysis with the differences in transcription factors was performed by the R packages ‘cluster Profiler’, ‘ggplot2’, ‘enrichplot’, ‘org. Hs. Eg. Db’, and ‘GOplot’ [24]. Finally, the ‘circlize’ [25] and ‘RColorBrewer’ [26] R packages were used to enrich the biological process (BP) of GO. Finally, PPI networks of TFs and key biomarkers were constructed with a minimum required interaction score of 0.400.

### 2.8. Analysis of the infiltration of immune cells and the relationship with key biomarkers

We scored 28 immune cells for each sample in GSE108109 and GSE200828 and evaluated the degree of infiltration of each immune cell in our sample by single-sample gene set enrichment analysis (ssGSEA).

Violin plots were drawn by the ‘vioplot’ package of R, which was mainly applied to express the variation in the infiltration expression level of the immune cells among the control and FSGS groups. We estimated this link between the two core genes and immune-infiltrating cells with ‘tidyverse’ [27] and ‘reshape2’ [28]. Subsequently, we applied ‘ggplot2’ for data visualization.

### 2.9. Statistical analysis

Due to the data species and characteristics, differences were analyzed by the Mann–Whitney *U* test and Student’s *t* test for the categorical variables and the continuous variables, respectively. The two-sided test was considered statistically significant at *p* < 0.05.

## 3. Results

### 3.1. Distinction of the DEGs

**Figure 1.**
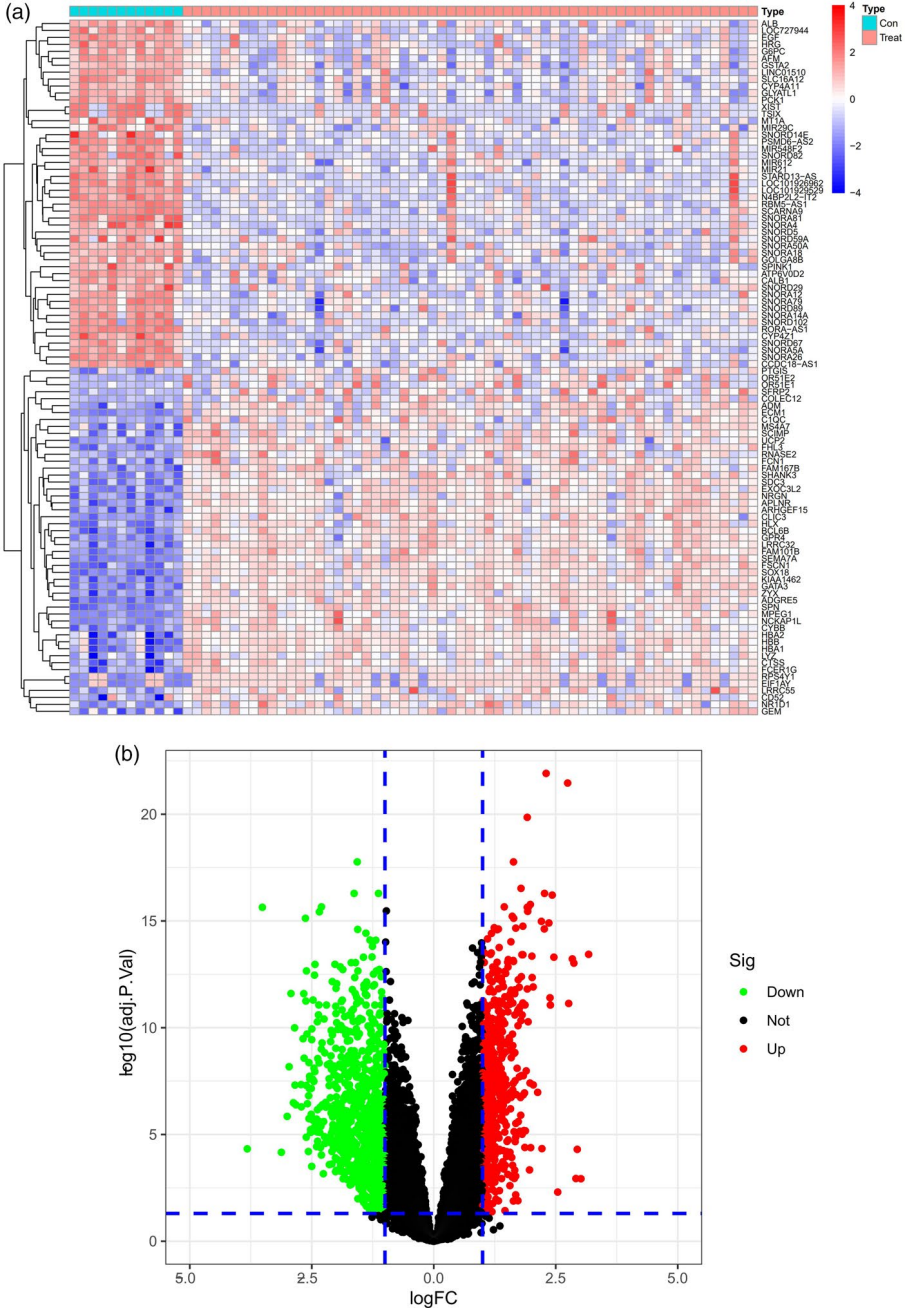
Identification of DEGs of the glomerulus of the FSGS and normal groups. (a) Heatmap. The horizontal axis shows a sample type, and the vertical axis displays the difference in expression between genomes. The top half indicated that the differential genes were up-regulated in the left control group and down-regulated in the right FSGS group. The bottom half was the opposite of the top half. (b) Volcano map. The horizontal axis represents the log FC, and the vertical axis represents -log10. A significant difference was defined as a *p* value <0.05. FSGS: focal segmental glomerulosclerosis; DEGs: differentially expressed genes.

A total of 1474 DEGs were identified, including 574 upregulated and 900 downregulated DEGs. A total of 1474 genes are shown in the heatmap (Figure 1(a)) and volcano map (Figure 1(b)).

### 3.2. DEG gene set enrichment analysis in FSGS

**Figure 2.**
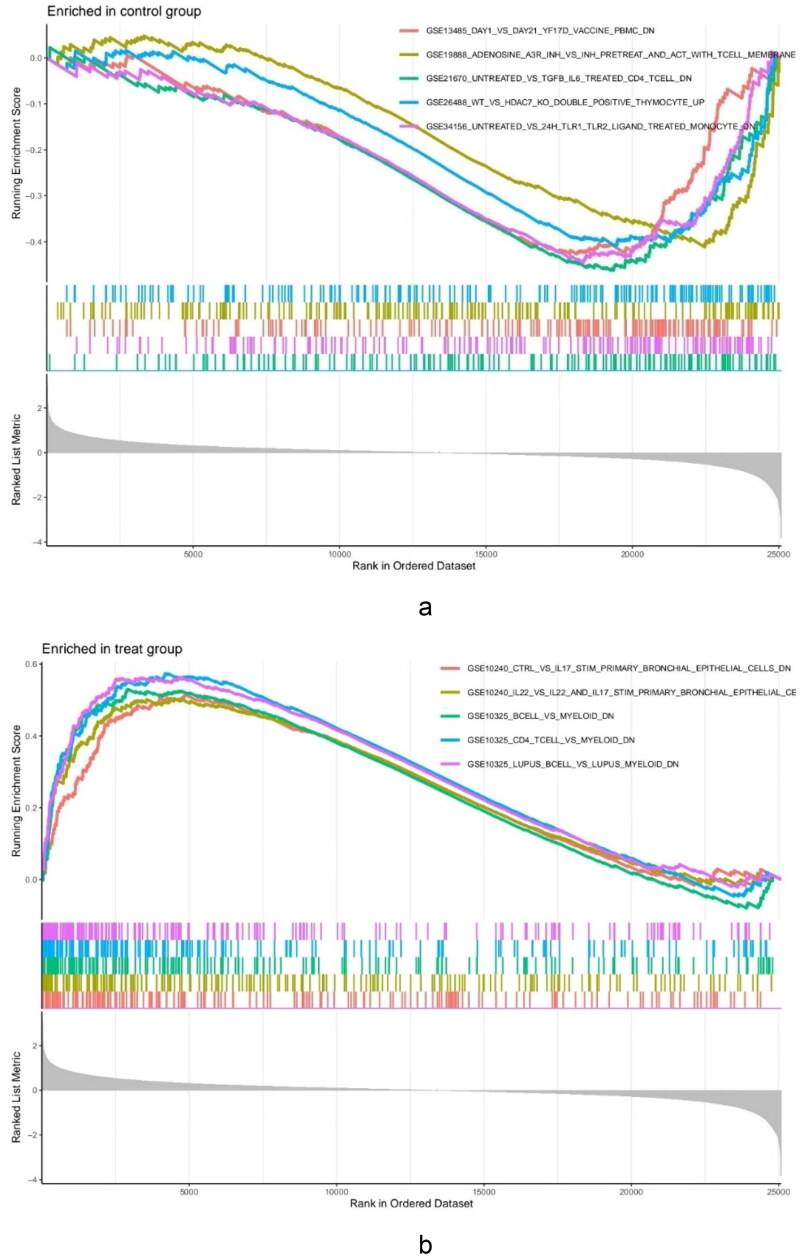
GSEA enrichment analysis for the DEGs. (a) Control groups. (b) The glomerulus of FSGS groups.

To explore the immune mechanism in the glomeruli of FSGS, the immune genes from the MSigDB database were used for GSEA of the DEGs (Table 2). Our findings found that the glomerulus of the FSGS group was particularly collected in CD4+ T cells, peripheral blood mononuclear cells (PBMCs), and B cells. The findings revealed the importance of genes related to immunity in the glomerulus of FSGS (Figure 2).

**Table 2.** GSEA enrichment analysis of DEGs.

ID	Enrichment score	NES	*p* Value	*p*. Adjust
GSE10240_CTRL_VS_IL17_STIM_PRIMARY_BRONCHIAL_EPITHELIAL_CELLS_DN	0.515659072	2.187832495	1.00E-10	3.35E-09
GSE10240_IL22_VS_IL22_AND_IL17_STIM_PRIMARY_BRONCHIAL_EPITHELIAL_CELLS_DN	0.504800764	2.140526883	1.00E-10	3.35E-09
GSE10325_BCELL_VS_MYELOID_DN	0.530238329	2.264947413	1.00E-10	3.35E-09
GSE10325_CD4_TCELL_VS_MYELOID_DN	0.573897613	2.436548064	1.00E-10	3.35E-09
GSE10325_LUPUS_BCELL_VS_LUPUS_MYELOID_DN	0.563279604	2.396822283	1.00E-10	3.35E-09
GSE10325_LUPUS_CD4_TCELL_VS_LUPUS_MYELOID_DN	0.572333261	2.43794543	1.00E-10	3.35E-09
GSE11057_CD4_CENT_MEM_VS_PBMC_DN	0.568902121	2.418033008	1.00E-10	3.35E-09
GSE11057_NAIVE_VS_EFF_MEMORY_CD4_TCELL_DN	0.508826463	2.163631833	1.00E-10	3.35E-09
GSE12845_IGD_NEG_BLOOD_VS_DARKZONE_GC_TONSIL_BCELL_UP	0.513330635	2.181834758	1.00E-10	3.35E-09
GSE13485_CTRL_VS_DAY7_YF17D_VACCINE_PBMC_DN	0.608126822	2.590413854	1.00E-10	3.35E-09
GSE13485_DAY7_VS_DAY21_YF17D_VACCINE_PBMC_UP	0.546486619	2.323770346	1.00E-10	3.35E-09
GSE13485_PRE_VS_POST_YF17D_VACCINATION_PBMC_DN	0.517638738	2.209285463	1.00E-10	3.35E-09
GSE1432_CTRL_VS_IFNG_24H_MICROGLIA_DN	0.531287361	2.252839064	1.00E-10	3.35E-09
GSE15930_STIM_VS_STIM_AND_IFNAB_48H_CD8_T_CELL_UP	0.526430211	2.246807526	1.00E-10	3.35E-09
GSE15930_STIM_VS_STIM_AND_IL12_48H_CD8_T_CELL_UP	0.541239017	2.311937567	1.00E-10	3.35E-09
GSE16266_CTRL_VS_LPS_STIM_MEF_UP	0.536987624	2.277010117	1.00E-10	3.35E-09
GSE16385_MONOCYTE_VS_12H_IL4_TREATED_MACROPHAGE_DN	0.590606499	2.504372378	1.00E-10	3.35E-09

### 3.3. PPI network and submodule analysis

To investigate the associations among the 1474 differentially expressed genes, we constructed their PPI networks. The network was built with the help of the STRING database. The network screening criterion was that the confidence of the connected nodes was greater than 0.4. The network contained 962 nodes and 5531 edges.

The five most significant modules (122 genes) were identified by the PPI network using the MCODE plugin (Table 3). Then, cytoHubba was used to mark the core gene in five modules. The ‘MCC’ algorithm was used to choose nine core genes (Figure 3(a)). The results indicated that CD4, tumor necrosis factor (TNF), tyrosine protein tyrosine kinase binding protein (TYROBP), epidermal growth factor (EGF), tumor suppressor p53 (TP53), Notch homolog 1 (NOTCH1), type 2 taste receptor 4 (TAS2R4), mucin 15 (MUC15), and alanine glyoxylate aminotransferase (AGXT) benefit the glomerulus in FSGS.

Figure 3. MCODE module analysis. Based on the PPI network constructed by the differentially expressed genes, five modules were identified by the MCODE plug-in. Each module consists of three parts. (a) Module information. Nodes in the network represented genes or proteins, and the lines between nodes represented interactions between them. One or three nodes with different colors in each module represented the core genes of the network. (b) Bubble map of module GO. The enrichment generation of each module was on the X-axis, and the name and its function were on the Y-axis. (c) Bubble map of module KEGG. The X-axis represented the enrichment rate of each module, and the Y-axis represents each function. MCODE: molecular complex detection.

**Figure:**
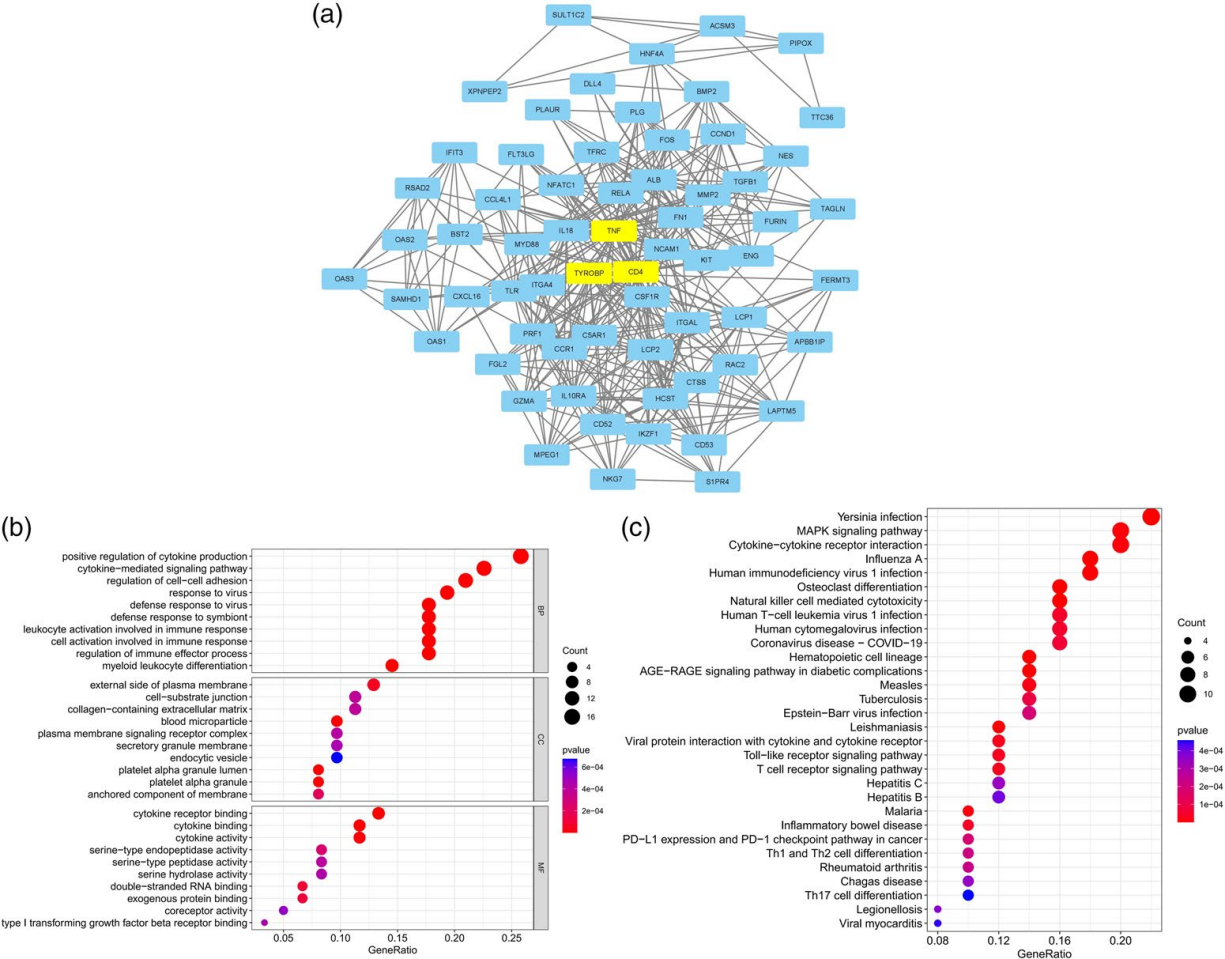

**Figure:**
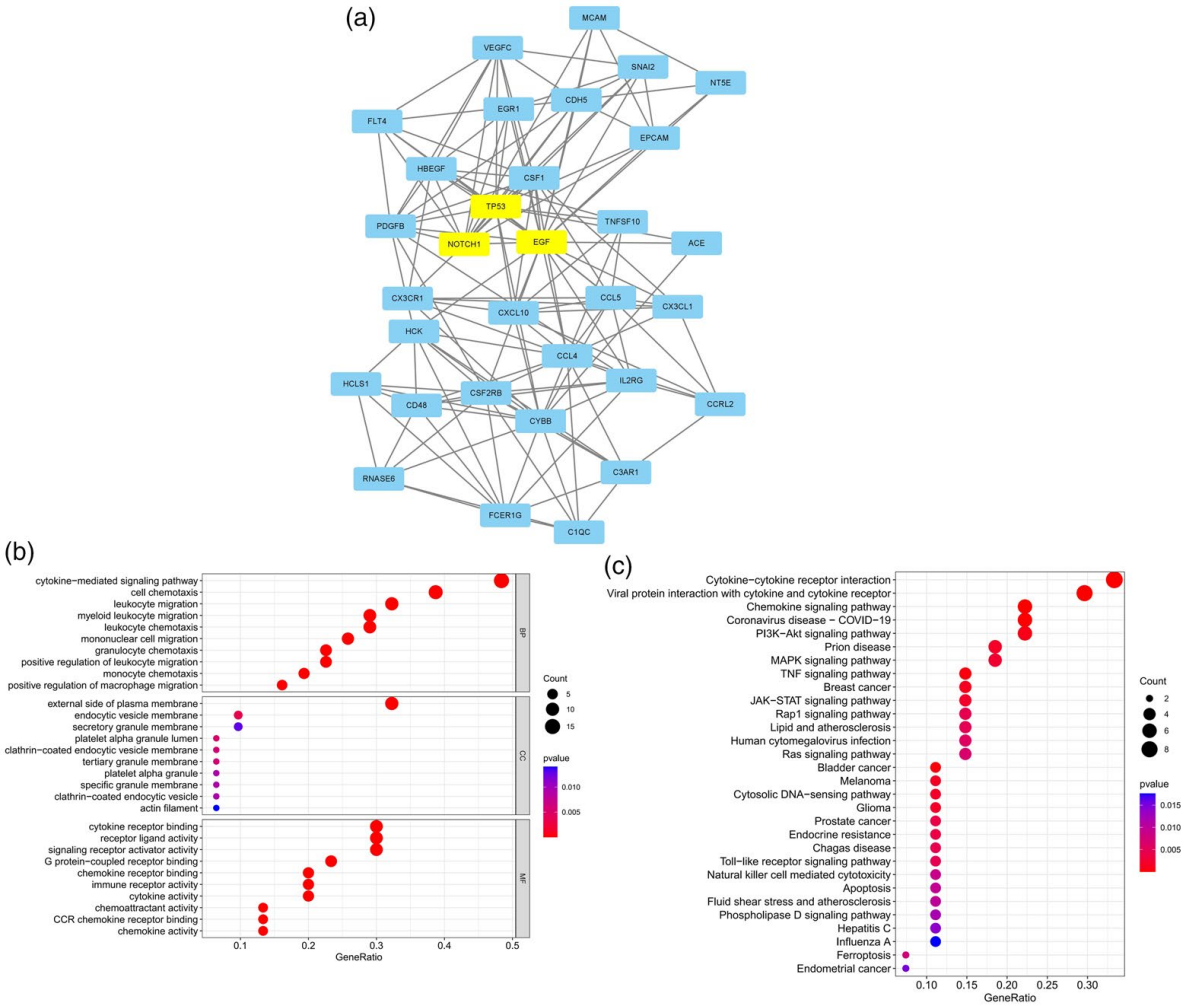

**Figure:**
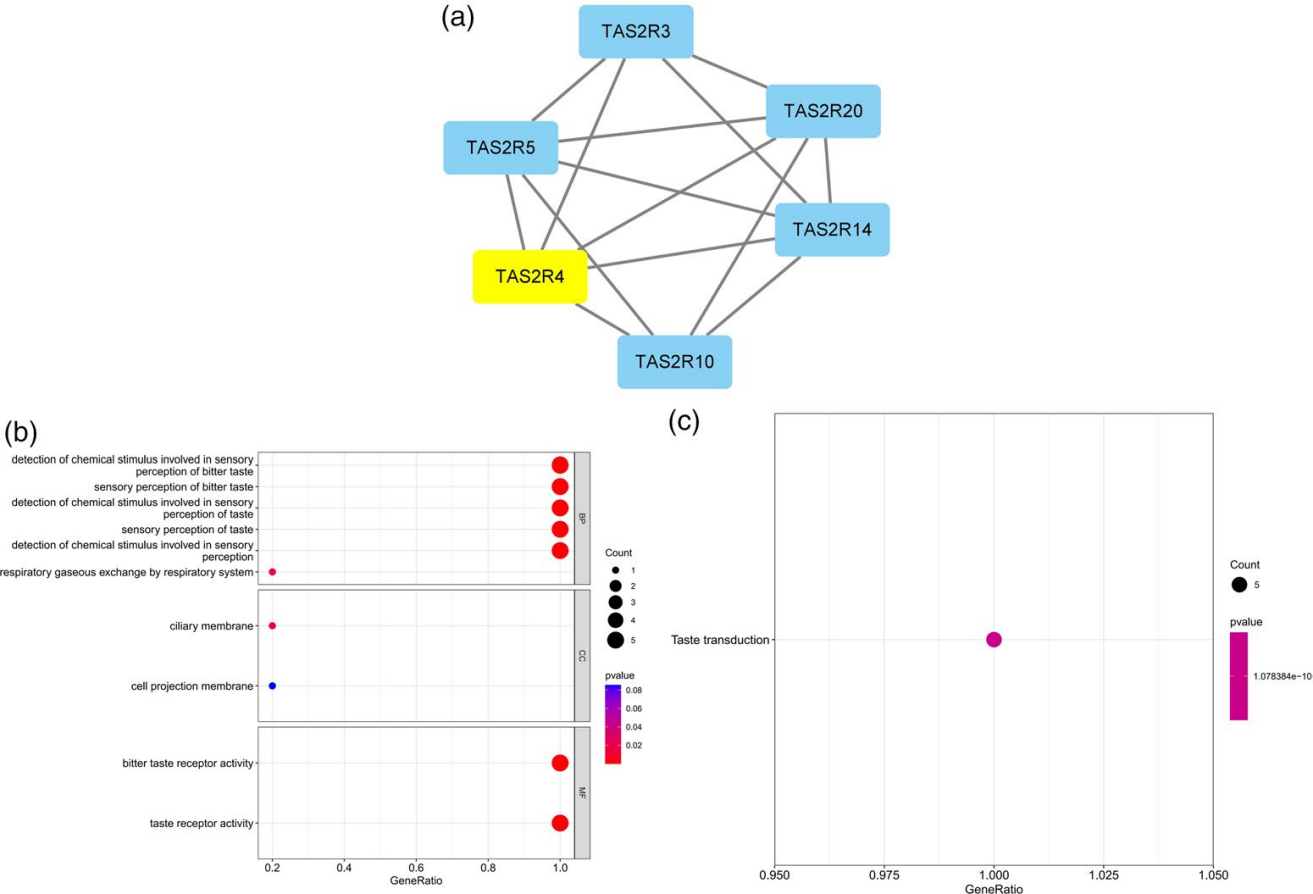

**Figure:**
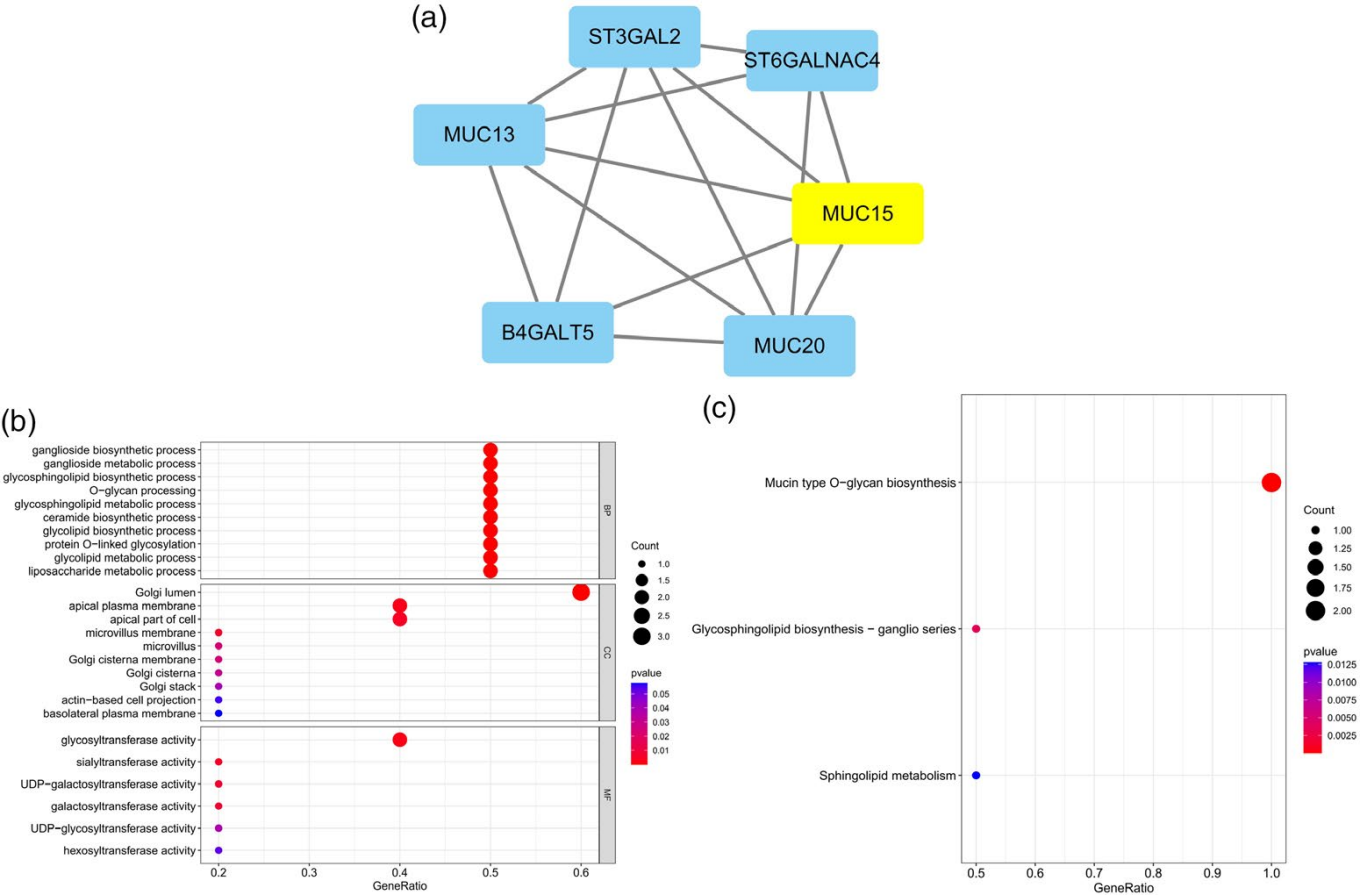

**Figure:**
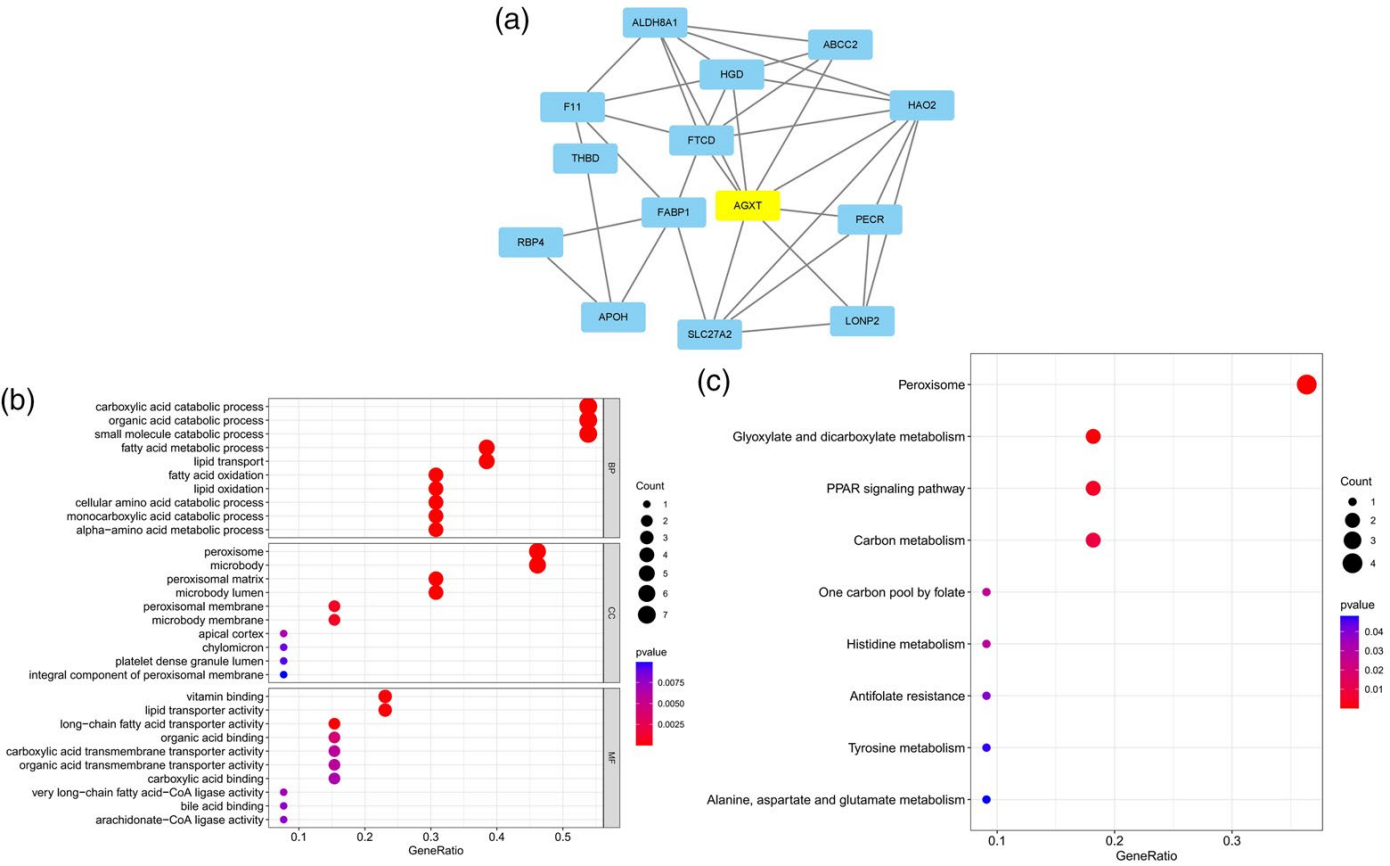

**Table 3.** Modules and key genes identified by MCODE.

Cluster	Score	Nodes	Edges	Node IDs
1	13.968	64	440	OAS1, FURIN, IL18, ITGA4, LCP1, TFRC, MPEG1, IKZF1, TAGLN, XPNPEP2, TYROBP, TNF, SULT1C2, FERMT3, PIPOX, PLG, IL10RA, HNF4A, FN1, TTC36, SAMHD1, BST2, CD52, ACSM3, FOS, NCAM1, CCND1, ALB, MYD88, CXCL16, HCST, RSAD2, OAS2, NKG7, IFIT3, LAPTM5, TGFB1, NFATC1, CD53, RELA, MMP2, TLR7, BMP2, RAC2, CD4, FLT3LG, CTSS, APBB1IP, NES, OAS3, PRF1, GZMA, FGL2, ITGAL, CSF1R, CCL4L1, S1PR4, ENG, PLAUR, LCP2, C5AR1, DLL4, CCR1, KIT
2	9.226	32	143	VEGFC, SNAI2, CSF2RB, CX3CL1, HCK, CCRL2, HCLS1, C3AR1, RNASE6, PDGFB, MCAM, FCER1G, EPCAM, ACE, FLT4, CYBB, CSF1, C1QC, NT5E, CD48, HBEGF, CCL4, CX3CR1, TNFSF10, IL2RG, EGR1, CDH5, CCL5, NOTCH1, TP53, CXCL10, EGF
3	5.6	6	14	TAS2R5, TAS2R14, TAS2R20, TAS2R10, TAS2R3, TAS2R4
4	5.6	6	14	ST3GAL2, B4GALT5, MUC20, MUC13, ST6GALNAC4, MUC15
5	5.231	14	34	F11, PECR, AGXT, LONP2, THBD, FABP1, FTCD, RBP4, ABCC2, HGD, ALDH8A1, SLC27A2, APOH, HAO2

GO and KEGG analyses were performed for each of our five modules based on MCODE. The results indicated that the core module GO was mostly enriched in the positive regulation of cytokine production, leukocyte activation involved in the immune response, cell activation involved in the immune response, myeloid leukocyte migration, leukocyte chemotaxis, leukocyte migration, positive regulation of macrophage migration, and mononuclear cell migration (Figure 3(b)).

KEGG analysis indicated that the core module was mostly enriched in the MAPK signaling pathway, cytokine–cytokine receptor interaction, chemokine signaling pathway, and TNF signaling pathway (Figure 3(c)).

### 3.4. Coexpression network construction and hub module identification

**Figure 4.**
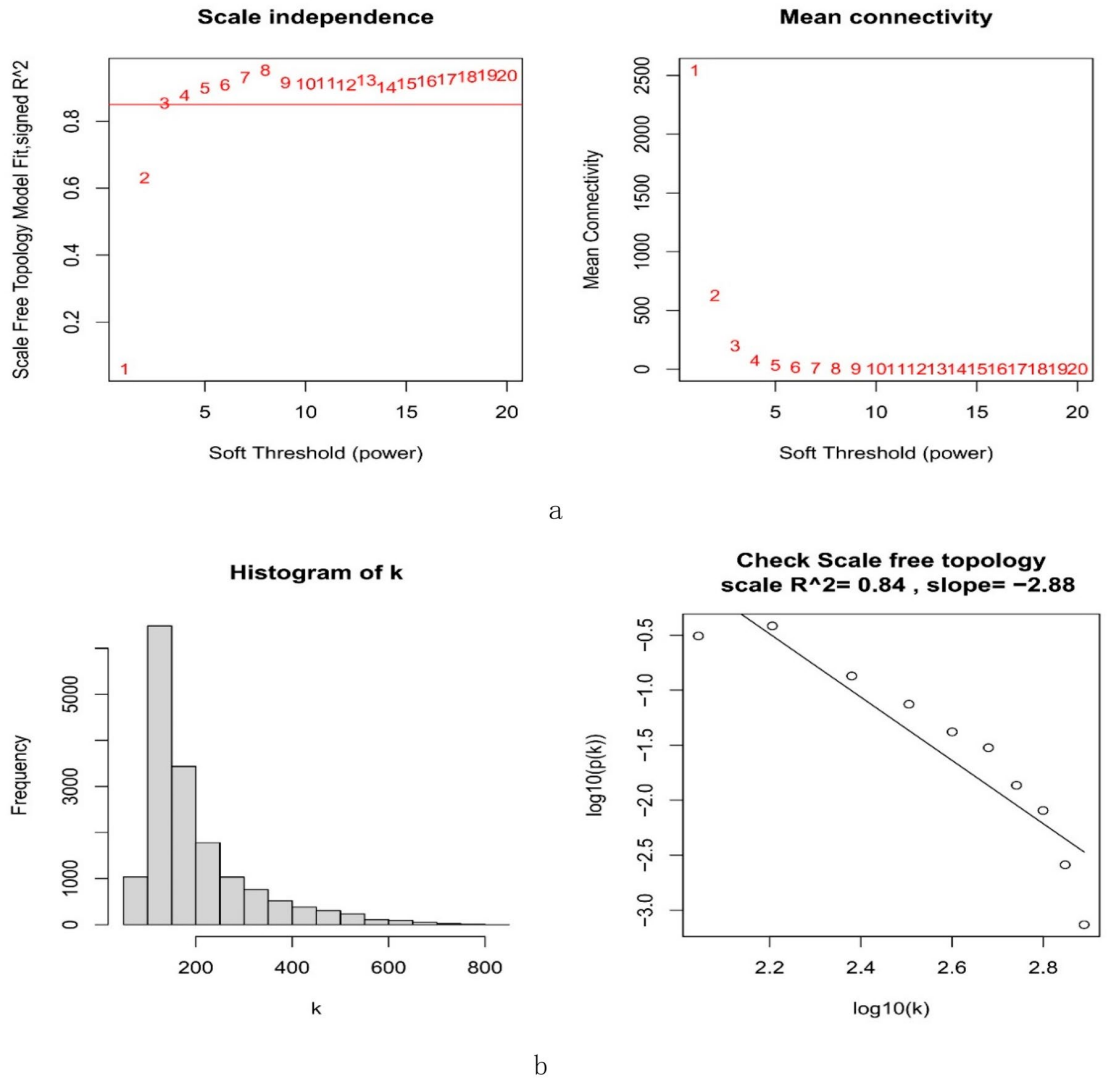
The decision for the soft-thresholding power (*β*) of WGCNA. (a) Analysis of the scale-free fit index and the mean connectivity. The line shows that the relations coefficient was 0.85, and this counterpart (*β*) was 3. (b) Histogram of connectivity distribution and evaluating the scale-free topology when *β* = 3. WGCNA: weighted gene coexpression network analysis.

**Figure 5.**
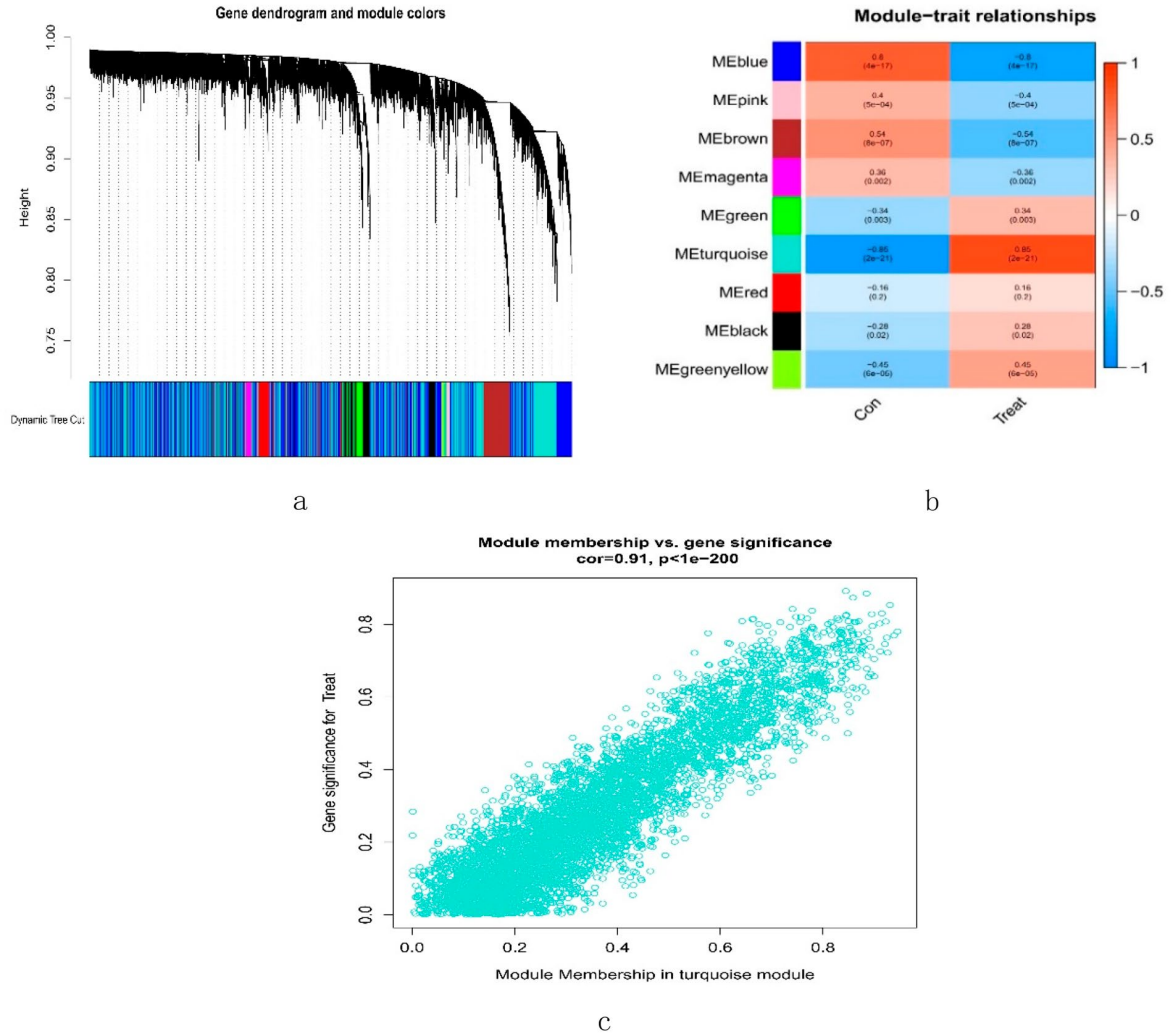
Creation of WGCNA modules. (a) A clustering tree of genes. The branches at the top of the image represented genes, and the rectangles at the bottom of the image were made up of various modules. (b) A chart for the module-trait relationship. The turquoise module was strongly linked to the glomerulus of FSGS. (c) Scatter plot for the correlation between module − trait connections and gene significance in the turquoise module. FSGS: focal segment glomerulosclerosis; WGCNA: weighted gene coexpression network analysis.

A total of 16,262 genes were chosen for WGCNA. We set the soft threshold to 3 (*R*^2^ = 0.84) to create a scale-free network (Figure 4(a,b)). A coexpression matrix was built, and nine modules were obtained by dynamic splicing (Figure 5(a)). The turquoise module (138 genes) had the greatest association with FSGS glomeruli (cor =0.85; p = 2e-21) (Figure 5(b)). There was a close relationship between module membership and gene significance in the turquoise module (cor = 0.91; p < 1e-200) (Figure 5(c)). Therefore, this turquoise module was selected for the next analysis.

### 3.5. Identification of hub genes

**Figure 6.**
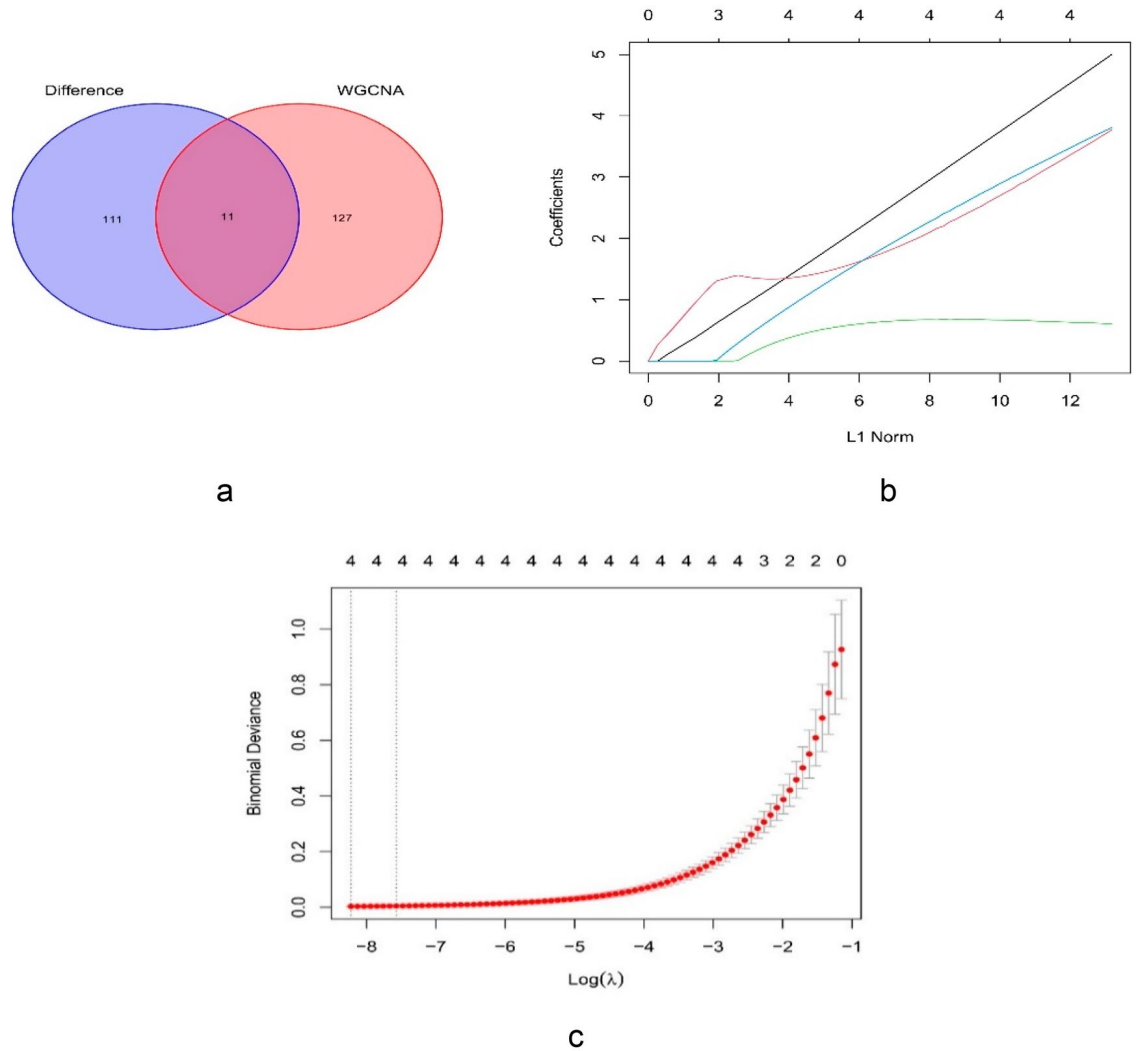
Identification of key genes. (a) Venn diagram for the cross genes using MCODE and WGCNA. (b) LASSO parameters of TGFB1, ENG, SNAI2, and NOTCH1 in FSGS. (c) The log (lambda) order was provided to create a parameter diagram. LASSO: least absolute shrinkage and selection operator.

The plug-in MCODE was employed to identify the 122 core genes. The eleven cross genes were collected from the 122 key genes of MCODE, and the 138 genes identified by WGCNA included FURIN, cyclin D1 (CCND1), transforming growth factor beta 1 (TGFB1), RELA (p65), nuclear export signal (NES), etonogestrel (ENG), delta-like 4 (DLL4), snail family transcriptional repressor 2 (SNAI2), Fms-like tyrosine kinase 4 (FLT4), vascular-endothelial [VE]–cadherin/CD144 (CDH5), and NOTCH1 (Figure 6(a)). After LASSO analysis of the genes, TGFB1, ENG, SNAI2, and NOTCH1 were identified as hub genes (Figure 6(b,c)).

### 3.6. Diagnostic value of hub gene expression levels

**Figure 7.**
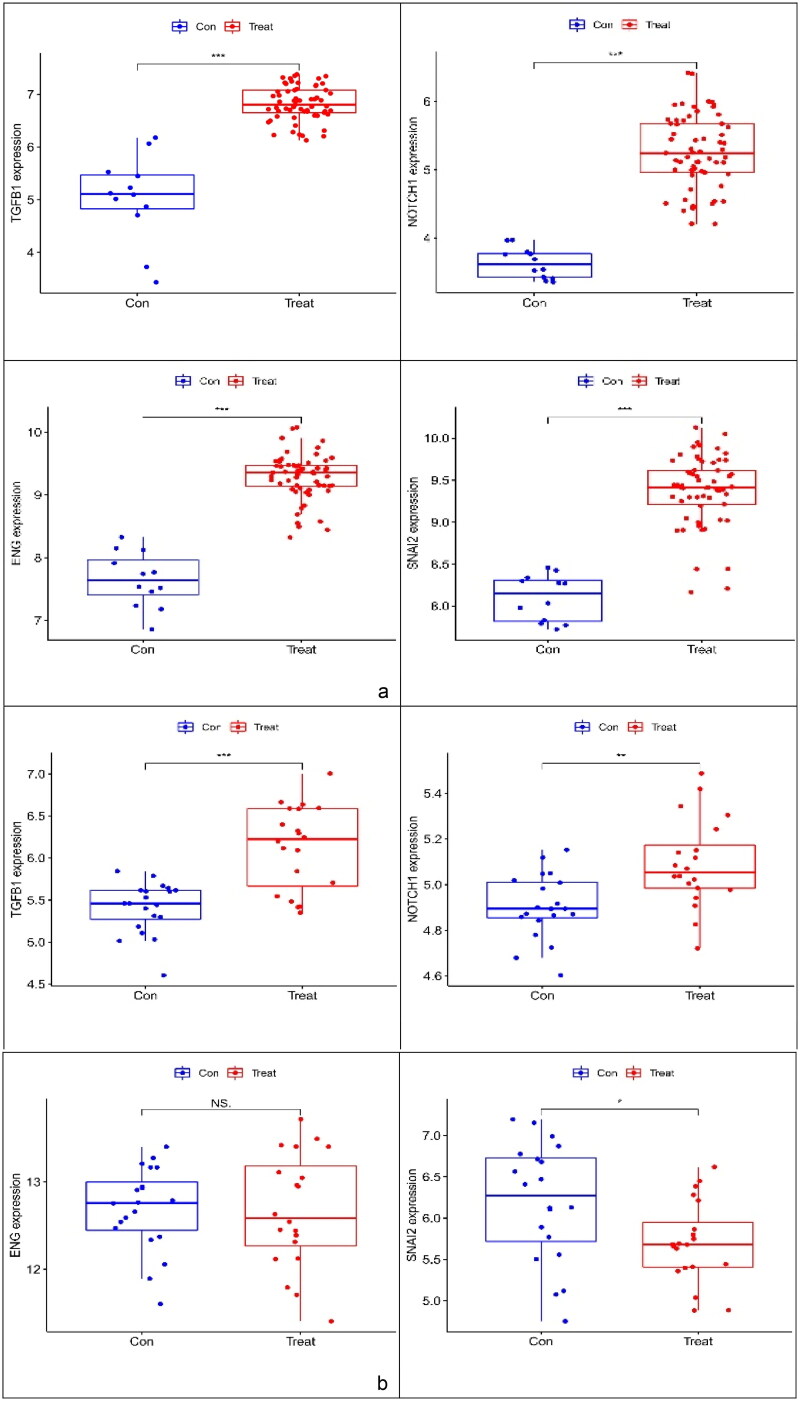
Boxplot of ENG, NOTCH1, TGFB1 and SNAI2. (a) Evaluation of four key genes in GSE108109 and GSE200828. The expression levels of ENG, NOTCH1, TGFB1 and SNAI2 were significantly higher in the glomerulus of FSGS than in that of the control group. (b) Evaluation of ENG, NOTCH1, and TGFB1 in GSE129973. The results were similar to those of GSE108109 and GSE200828. However, the SNAI2 gene was expressed at lower levels in the glomerulus of FSGS compared with that in the control group. FSGS: focal segmental glomerulosclerosis.

The expression levels of TGFB1, ENG, SNAI2 and NOTCH1 were verified using boxplots. TGFB1, ENG, SNAI2 and NOTCH1 expression levels (*p* < 0.001) were greater in the glomerulus of FSGS than in that of the control group (Figure 7(a)). A dataset (GSE129973) was applied to verify that the expression of the three markers (TGFB1, ENG and NOTCH1) was consistent with that in GSE108109 and GSE200828, and the expression pattern of SNAI2 was opposite of that of the previous trend (Figure 7(b)).

**Figure 8.**
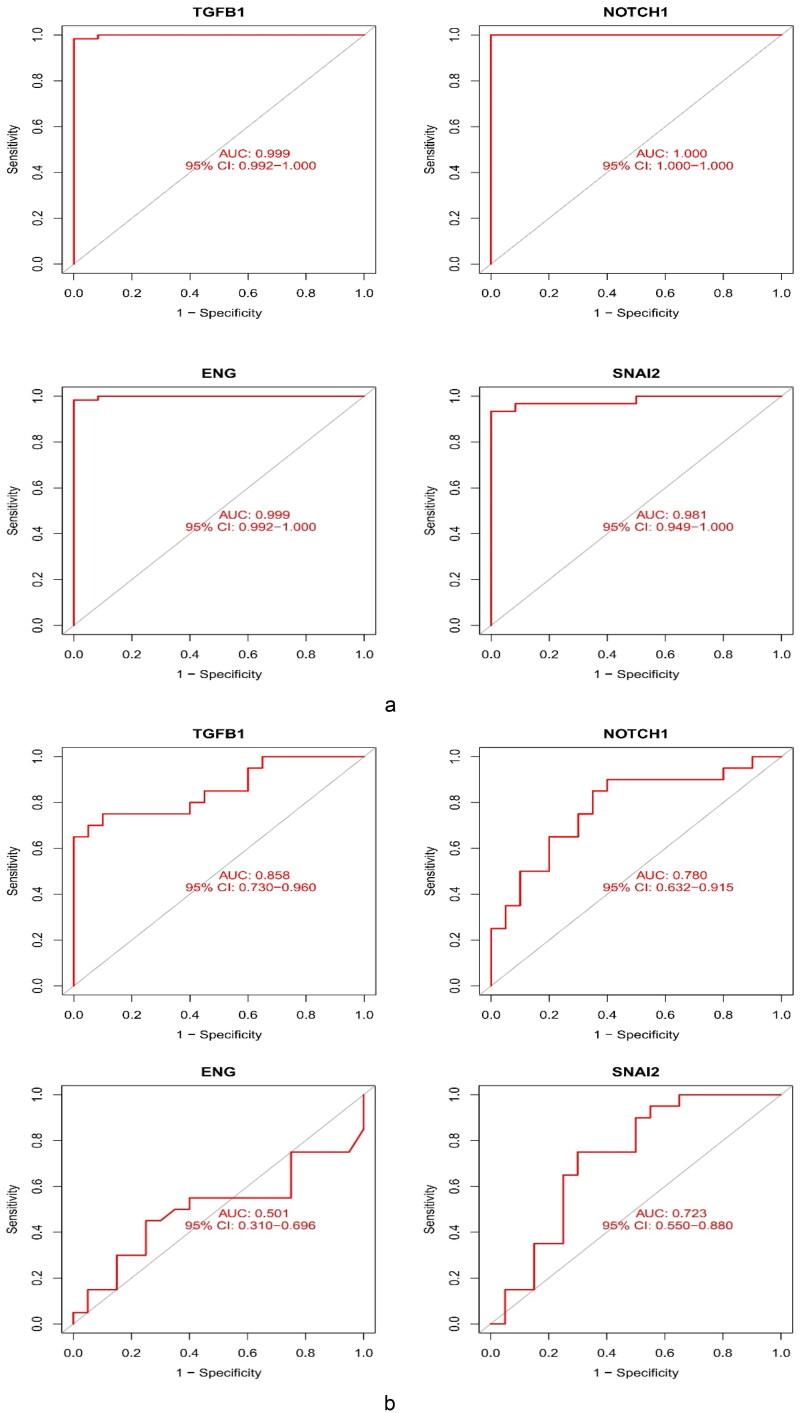
Validation of the diagnostic value of the hub genes. (a) Evaluation of the key genes of GSE108109 and GSE200828. ROC curves with AUC were provided to verify the ability to differentiate the glomerulus of FSGS from that of the control group based on sensitivity and specificity. (b) Evaluation of TGFB1 and NOTCH1 in GSE129973. These results were similar to those of GSE108109 and GSE200828. ROC: receiver operating characteristic; AUC: area under the curve.

To assess the diagnostic accuracy of the four core genes, we used ROC curves and their AUC values. The AUC values of the four genes were >0.75. The core genes had great diagnostic v™alue f™or FSGS (Figure 8(a)). Subsequently, we used the external dataset GSE129973 to verify the diagnostic value of the four key markers (Figure 8(b)).

Finally, TGFB1 and NOTCH1 were identified as potential key biomarkers.

### 3.7. TF regulatory network of TGFB1 and NOTCH1

**Figure 9.**
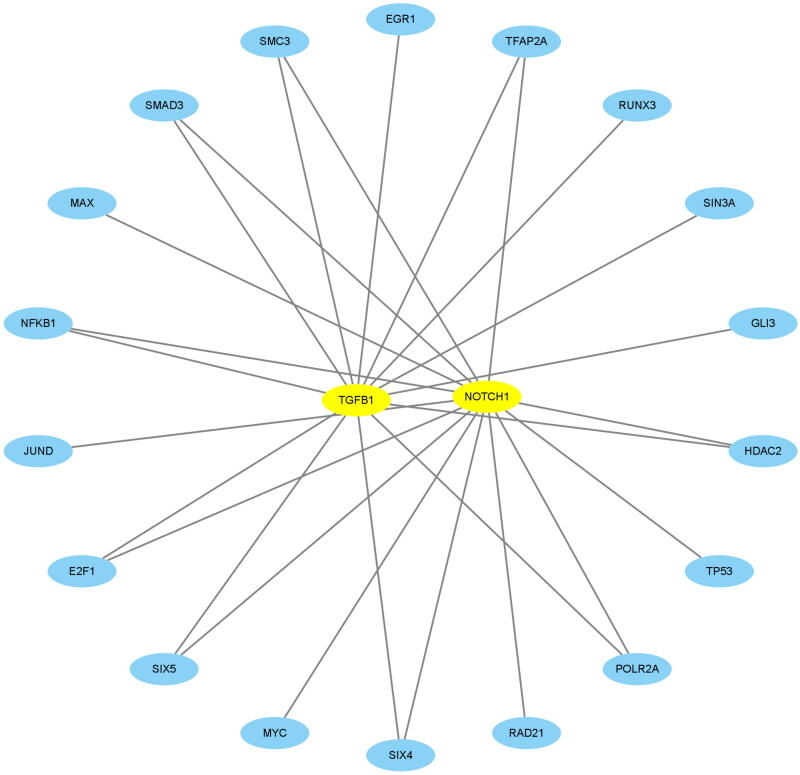
Construction of the network of transcription factors. The blue ellipse represents the transcription factors. The circle was made up of transcription factors. TGFB1 and NOTCH1 in the middle represented key biomarkers. The lines connecting the two nodes represent the regulation of TGFB1 and NOTCH1 by the transcription factors.

Using NES >4.0, we identified 18 TFs that were highly correlated with TGFB1 and NOTCH1 and plotted a network regulation chart of them (Table 4, Figure 9).

**Table 4.** Transcription factors of TGFB1 and NOTCH1.

Rank	AUC	NES	Transcription factor	Target genes
1	0.896861	8.71999	SMC3	TGFB1,NOTCH1
3	0.826607	8.01648	NFKB1	NOTCH1,TGFB1
4	0.65994	6.34752	SMAD3	TGFB1,NOTCH1
5	0.610613	5.85356	HDAC2	NOTCH1,TGFB1
4	0.676383	5.11975	E2F1	TGFB1,NOTCH1
6	0.494768	4.69352	TP53	NOTCH1
7	0.494021	4.68604	POLR2A	NOTCH1
8	0.486547	4.6112	MAX	NOTCH1
9	0.483558	4.58126	EGR1	TGFB1
10	0.476084	4.50642	NFKB1	TGFB1,NOTCH1
11	0.474589	4.49145	EGR1	TGFB1
12	0.473842	4.48397	EGR1	TGFB1
14	0.461883	4.36422	GLI3	TGFB1
5	0.571001	4.2599	SIX4,SIX5	NOTCH1,TGFB1
16	0.45142	4.25944	HDAC2	NOTCH1
15	0.45142	4.25944	POLR2A	TGFB1
17	0.44843	4.22951	JUND	NOTCH1
18	0.446188	4.20705	EGR1	TGFB1
19	0.443946	4.1846	RAD21	NOTCH1
20	0.440957	4.15467	MYC	NOTCH1
21	0.435725	4.10228	NFKB1	TGFB1,NOTCH1
22	0.434978	4.09479	SIN3A	TGFB1
23	0.433483	4.07982	RUNX3	TGFB1
25	0.426009	4.00498	TFAP2A	TGFB1,NOTCH1

**Figure 10.**
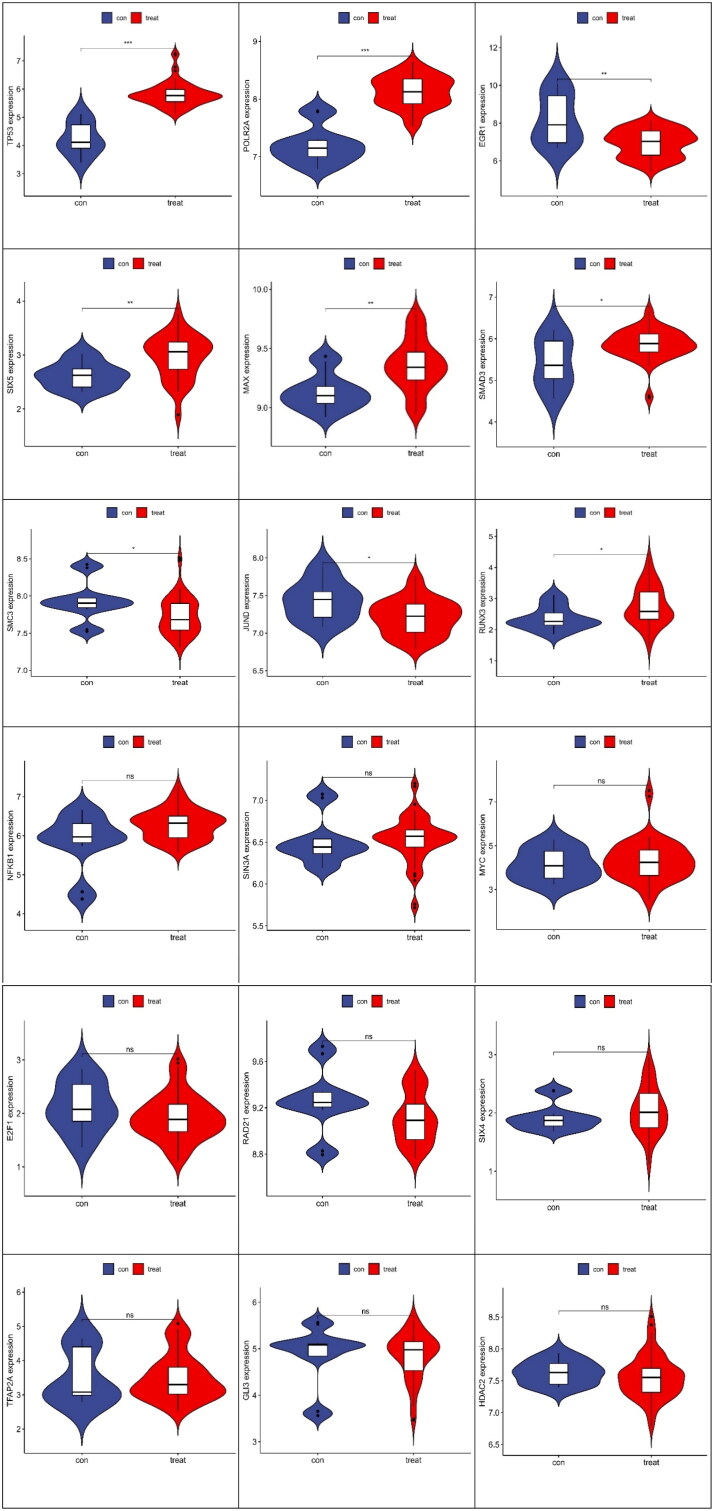
Differential analysis of transcription factors between the control group and FSGS group. The horizontal axis represents the control and FSGS groups, the vertical axis represents the expression of transcription factors. In each picture, the figure on the left represented the control group and the figure on the right represented the FSGS group. ***Represents *p* less than 0.001; **represents *p* less than 0.01; *indicates *p* less than 0. 05.

As seen from the above TF network diagram, 18 TFs were screened. Moreover, to clarify the differential expression of 18 transcription factors in the samples of our study with the control and FSGS groups, 9 transcription factors with significant differences were obtained (Figure 10).

**Figure 11.**
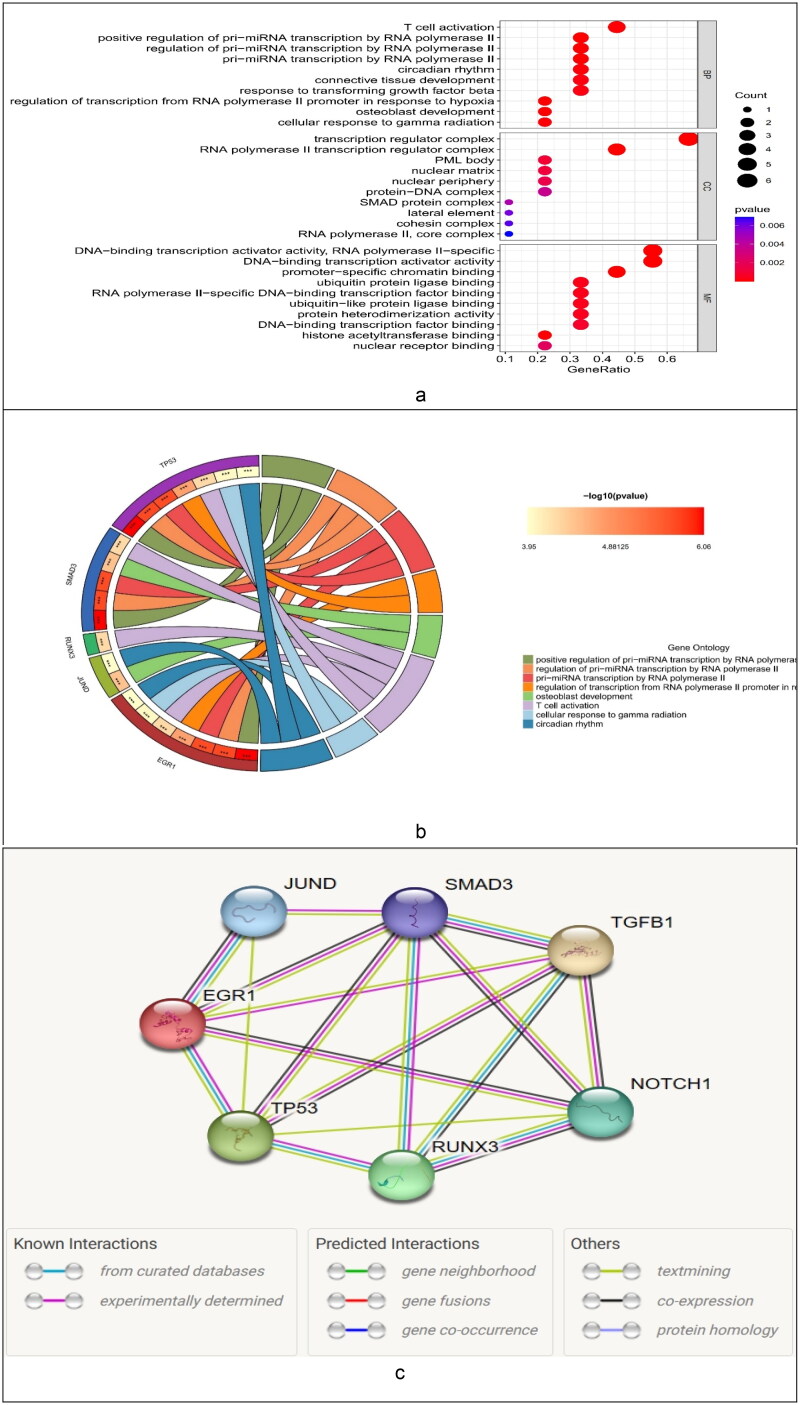
GO function analysis. (a) Bubble chart. (b) GOcircos. The outermost part of the left semicircle of the circle was the name of the transcription factor. The asterisk indicates a significant difference, and ***indicates *p* < 0.001. The right semicircle represents the top 8 functional features that were significantly enriched in GO. The band in the middle represents the association between transcription factors and functions. (c) PPI network relationships between transcription factors and target genes. The lower legend colors represent the relationship between them.

We obtained 9 differential TFs. To elucidate the functional characteristics of these TFs, we performed GO functional analysis (Figure 11(a,b)).

To clarify the relations between the TFs and target genes, we employed the STRING online database to construct their interaction relationship (Figure 11(c)).

### 3.8. Immune cell infiltration analysis and its relationship with NOTCH 1 and TGFB1

**Figure 12.**
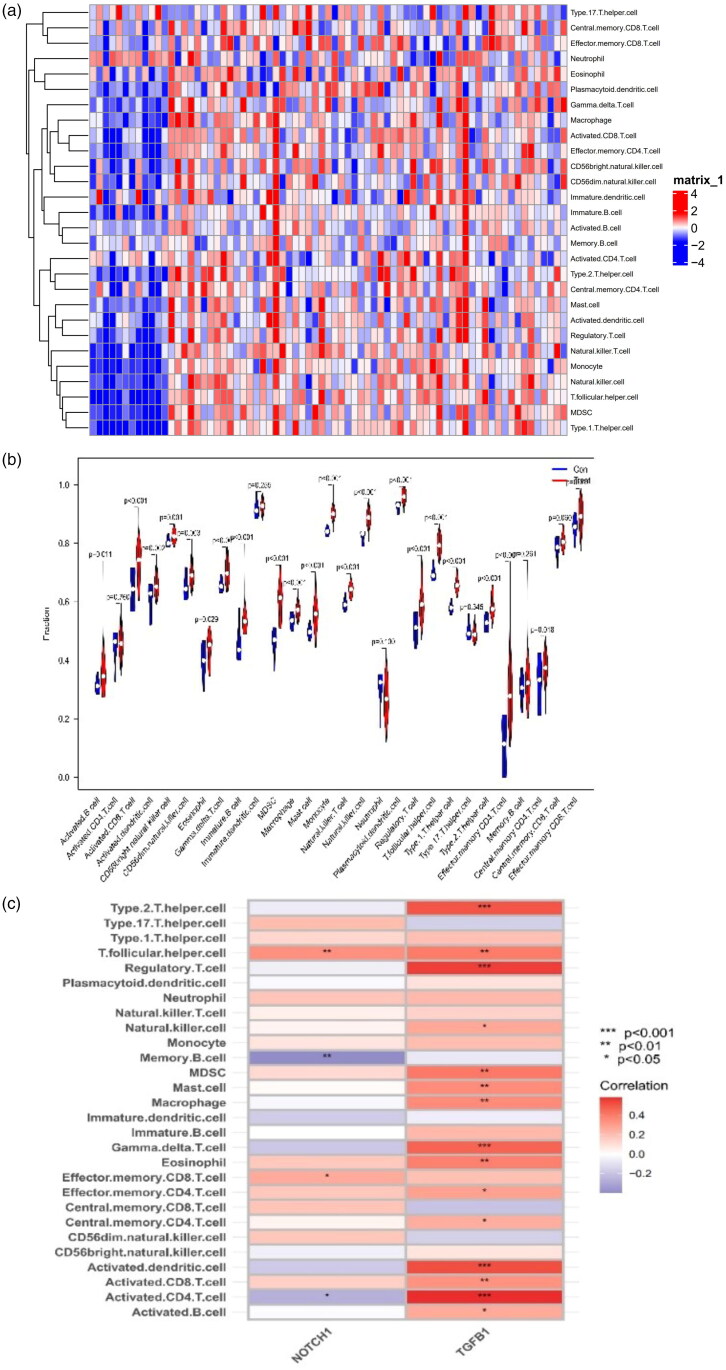
Analysis of immune-related interactions in the glomerulus of focal segmental glomerulosclerosis. (a) Heatmap. (b) Violin plot. (c) The link between two key biomarkers and immune cell infiltration.

We performed ssGSEA to obtain scores for the immune cells of each sample in the control group and glomerular FSGS group; these are presented as files (Table 5). The heatmaps of immune cells in the GSE108109 and GSE200828 datasets are presented in Figure 12(a). The infiltration levels of type 1 T helper cells, MDSCs, monocytes, natural killer cells, T follicular helper cells, natural killer T cells, and type 2 T helper cells in the glomerulus of the FSGS group were mostly higher than those in the control group. These results indicate that the immune cells had infiltrated the glomerulus of FSGS (Figure 12(b)). The relationships between the 28 immune cells and the key biomarkers suggested close interactions between NOTCH 1 and T follicular helper cells, memory B cells (*p* < 0.01), effector memory CD8 T cells, and activated CD8 T cells (*p* < 0.05) and interactions between TGFB1 and type 1 T helper cells, regulatory T cells, gamma delta T cells, activated dendritic cells, and activated CD4 T cells (*p* < 0.001) (Figure 12(c)).

**Table 5.** Infiltration of 28 immune cells.

Immune cell	*p* Value
Type.1.T.helper.cell	5.33E-08
MDSC	6.84E-08
T.follicular.helper.cell	8.06E-08
Monocyte	1.97E-07
Natural.killer.cell	1.97E-07
Natural.killer.T.cell	1.47E-06
Type.2.T.helper.cell	1.84E-06
Effector.memory.CD4.T.cell	1.98E-06
Regulatory.T.cell	1.75E-05
Plasmacytoid.dendritic.cell	2.14E-05
Macrophage	5.67E-05
Gamma.delta.T.cell	8.26E-05
Mast.cell	9.34E-05
Activated.CD8.T.cell	0.00019267
Immature.B.cell	0.000204369
CD56bright.natural.killer.cell	0.001086804
Activated.dendritic.cell	0.001644126
CD56dim.natural.killer.cell	0.002578219
Activated.B.cell	0.011157739
Effector.memory.CD8.T.cell	0.011157739
Central.memory.CD4.T.cell	0.01759676
Eosinophil	0.029220138

*p* Value < 0.05

## 4. Discussion

FSGS is a clinicopathologic disorder that is characterized by segmental and localized glomerular injury, macroalbuminuria, nephrotic syndrome (NS), and primary resistance to hormonal therapy [29]. The incidence of FSGS is closely related to the injury and loss of podocytes [30], and its production and development are strongly linked to the immune response [31]. Due to limited diagnostic techniques and therapeutic methods, clinical cure rates are generally lower, and the prognosis is poorer. Therefore, there is a need to understand the underlying mechanisms of FSGS. In particular, the study of infiltrating immune cells in glomerular tissue are needed for the identification of new differential diagnostic markers for the diagnosis and treatment of FSGS.

In this study, we downloaded two gene chips, GSE108109 and GSE200828, from the GEO dataset for a series of bioinformatics analyses. First, 1474 DEGs were obtained, of which 574 were upregulated and 900 were downregulated. A PPI network for the DEGs was generated using the MCODE plugin of Cytoscape software, and subsequently, five core modules, including 122 core genes, were obtained. GO and KEGG functional analyses of the 122 core genes suggested that the expression changes of these DEGs affected signal transduction pathways related to inflammation and the immune response, such as the MAPK signaling pathway and cytokine-mediated signaling pathway. Additionally, inflammation was also one of the more common pathways. For example, Hongtian Wang [32] et al. demonstrated through animal and clinical studies that fibrinogen is associated with podocyte injury through the TLR4-p38 MAPK-NF-κB p65 pathway and is associated with disease activity in FSGS patients. Recent clinical studies suggested that inhibition of p38 MAPK with losmapimod could not reduce albuminuria by ≥50% in FSGS patients. However, it has been suggested that p38 MAPK inhibition may have strong therapeutic potential for FSGS patients in the future [33]. Interleukin-1β, tumor necrosis factor-α (TNF-α) and transforming growth factor-β1 (TGF-β1) have become important factors in the occurrence and development of FSGS [6]. These results are in accordance with the results from the functional enrichment analysis in our study. The enrichment results may be associated with chronic renal disease linked with albuminuria and progressive glomerular injury.

To better understand the underlying mechanisms of FSGS, candidate biomarkers for FSGS were identified using WGCNA in this study. Using WGCNA, we obtained nine modules, of which the turquoise module (138 genes) was most highly associated with glomerular FSGS. We intercrossed the genes of the five modules of MCODE with those of the WGCNA turquoise module to obtain 11 hub genes (FURIN, CCND1, TGFB1, RELA, NES, ENG, DLL4, SNAI2, FLT4, CDH5, and NOTCH1), followed by four key genes (TGFB1, ENG, SNAI2, and NOTCH1) obtained by the lasso algorithm. Two key biomarkers, TGFB1 and NOTCH1, were finally obtained by box plot and ROC curve verification of the external dataset GSE129973.

Transforming growth factor-beta (TGF-beta) plays a key role in the development of focal segmental glomerulosclerosis [34]. TGF-β1 is a multifunctional cytokine with multiple roles in fibrosis and inflammation. It acts through the Smad signaling pathway in renal pathology and is closely associated with glomerulosclerosis [35]. Previous studies on this subject suggested that the protective effect of quercetin on adriamycin-induced glomerulosclerosis in rats mainly acts by altering the expression level of TGF-β signaling pathway-related proteins. For example, it can reduce the expression level of TGF-β1 protein and slow the progression of glomerulosclerosis [36]. Activation of the NOTCH signaling pathway is mostly linked to the occurrence and development of glomerular diseases. Dou Y et al. [37] used immortalized podocytes and adriamycin-induced FSGS mouse models to confirm that baicalin improves the FSGS process by reducing podocyte damage and revealed the potential mechanism by which baicalin inhibits the NOTCH1-snail axis-mediated podocyte epithelial-mesenchymal transition (EMT).These results support the key biomarkers screened in this study.

Studies have indicated that the dysfunction of transcription factors is greatly attributable to the pathogenesis or disease progression of FSGS [38,39]. To elucidate the mechanism of action of the two key biomarkers, we identified 18 transcription factors that may be associated with them. Subsequently, the differential expression of these 18 transcription factors between the control and glomerular FSGS groups was observed in our study, and 9 transcription factors (SMAD3, SIX5, POLR2A, RUNX3, SMC3, EGR1, TP53, JUND, and MAX) were obtained. Among them, TP53 and POLR2A had the greatest difference (*p* < 0.001). The biological process (BP) GO enrichment analysis was also performed, and the results suggested that it was closely related to the transcriptional regulation of pri-miRNA by RNA polymerase II, the development of osteoblasts, and T-cell activation. The involved transcription factors included TP53, Smad3, Runx3, Jund and EGR1. Through advances in cryo-electron microscopy and software analyses, the functional domain of P53 was found to directly regulate the DNA-binding activity of RNA polymerase II, thereby mediating transcription [40]. Wu J et al. [41] demonstrated through clinical studies on FSGS patients and *in vitro* and *in vivo* experiments that miR-30s protected podocytes by targeting NOTCH1 and P53, and the downregulation of miR-30s led to enhanced signal transduction of NOTCH1 and P53, leading to podocyte damage. Clinical studies have shown that TGF-β1, TSP-1 and TGF-Betaiir protein and mRNA expression levels, as well as phosphorylated Smad2/Smad3 levels, are significantly elevated in FSGS cases [42]. Su Zeyu et al. [43] provided new insights into the pivotal role of CLEC14A in maintaining podocyte function and suppressing NF-κB signaling and early growth response protein 1 (EGR1) signaling. These findings indicate that CLEC14A may be an innovative therapeutic target for FSGS. In conclusion, the transcription factors TP53, Smad3 and EGR1, which are related to key biomarkers, are strongly associated with the occurrence and development of FSGS. The above results are consistent with the enrichment results in this study. The roles of Runx3 and Jund in the mechanisms underlying FSGS need further study. Interestingly, T-cell activation was indicated in the BP enrichment analysis of the key biomarker transcription factors, suggesting that the key biomarkers may be closely related to T-cell activation. This result provides an important reference for the subsequent infiltration of immune cells.

GSEA enrichment analysis showed that CD4+ T cells ranked highly, followed by B cells. B cells can be modulated by CD4+ T cells and other factors, such as cytokines and TLR ligands [44]. CD4+ T cells and B cells are mostly related to the development of FSGS [45,46]. These results of studies are consistent with our results. These findings highlight the importance of adaptive immune cells in FSGS.

To determine the effects of immune cell infiltration on FSGS, we conducted a comprehensive assessment of FSGS immune infiltration using ssGSEA. The number of type 1 T helper cells, MDSCs, monocytes, natural killer cells, natural killer T cells, and T follicular helper cells were higher in FSGS samples than in control samples. Clinical studies have suggested that Th1 cytokines, especially IL-12, IL-2 and GM-CSF, may be involved in the pathology and progression of FSGS [47]. Limin Li et al. [48] demonstrated that glucocorticoid treatment ameliorates FSGS by expanding functional MDSCs [49]. A study demonstrated that pro-Th1 iNKT agonists can regulate a complex network to maintain podocyte physiology, providing a new approach for FSGS management [50]. The analysis of the associations between immune cells and the two key biomarkers showed activated CD4+ T cells, regulatory T cells, type 2 T helper cells, activated dendritic cells, T follicular helper cells, memory B cells and gamma delta T cells were associated with the two key biomarkers. Moreover, studies have shown that TGFB1 and NOTCH1 are associated with T cells [51,52]. Additionally, considering the results of the functional enrichment analysis of the transcription factors of the key biomarkers, this study suggested that T-cell infiltration (adaptive immune response) was highly related to glomerular FSGS lesions.

This study and the study by Zhu et al. [10] adopted the method of screening differential genes, constructing PPI networks, using MCODE and cytoHubba to screen hub genes, and identify the enriched transcription factors of the hub genes. The difference between these studies is that the study by Zhu et al. used different information to predict the hub genes of miRNAs and lncRNAs. However, in our study, WGCNA and LASSO were used to help identify hub genes, and GSEA and ssGSEA were used to analyze the relationships between key biomarkers and immune cell infiltration. To clarify the correlation between our key genes (TGFB1 and NOTCH1) and the key genes identified by Zhu et al. (C3AR1, CCR1, CX3CL1, MTNR1A, and P2RY13), we used the STRING database, and a confidence score >0.150 was used as a discrepancy. Then, a PPI network between them was constructed(see image below). In addition to MTNR1A, PPIs were found between the key genes. Among the key genes, TGFB1, NOTCH1, C3AR1, CCR1 and CX3CL1 were the most closely related. These genes are highly related to cytokine–cytokine receptor interactions, and studies have shown that they are related to T cells [53-55].Our research group previously identified the enriched hub nodes in the MAPK signaling pathway, suggesting that activated mast cells had a decisive influence on FSGS though renal tubule lesions and tubule interstitial inflammation. The PPI network was constructed between TGFB1, NOTCH1, DUSP1 and NR4A1, and a confidence level >0.400 was set as important. The results showed that there were interactions between these genes(see image below). TGF-β1 receptor blockade can prevent UUO-induced renal fibrosis by indirectly regulating the Smad and MAPK signaling pathways [56]. Overexpression of miR-499a leads to the inhibition of glioma cell proliferation and inhibition of the MAPK signaling pathway by reducing NOTCH1 to promote cell apoptosis [57]. In this study, the MAPK signaling pathway was still an enriched hub node, and T-cell immune infiltration was closely related to glomerular FSGS lesions. This study confirmed the interaction between T cells and mast cells [58]. In conclusion, this study suggests that the MAPK signaling pathway may play an important role in FSGS lesions, and the interaction between T cells and mast cells may indicate the immune regulatory mechanism of FSGS.

Our study had some limitations. First, the control and FSGS group samples in our study are still insufficient, which will affect the precision of the evaluation of the key biomarkers. Second, further experimental validation will be needed.

## 5. Conclusion

The infiltration of T cells is closely related to glomerular lesions in FSGS. TGF-β1 and NOTCH1 may play important roles in the glomeruli in FSGS through immune-related signaling pathways.
